# Lanthanum nitrate as aqueous electrolyte additive for favourable zinc metal electrodeposition

**DOI:** 10.1038/s41467-022-30939-8

**Published:** 2022-06-06

**Authors:** Ruirui Zhao, Haifeng Wang, Haoran Du, Ying Yang, Zhonghui Gao, Long Qie, Yunhui Huang

**Affiliations:** 1 Institute of New Energy for Vehicles, School of Materials Science and Engineering, Tongji University, Shanghai, 201804 China; 2 State Key Laboratory of Material Processing and Die & Mold Technology, School of Materials Science and Engineering, Huazhong University of Science and Technology, Wuhan, Hubei Province 430074 China

**Keywords:** Batteries, Batteries, Chemical physics, Materials for energy and catalysis, Materials science

## Abstract

Aqueous zinc batteries are appealing devices for cost-effective and environmentally sustainable energy storage. However, the zinc metal deposition at the anode strongly influences the battery cycle life and performance. To circumvent this issue, here we propose the use of lanthanum nitrate (La(NO_3_)_3_) as supporting salt for aqueous zinc sulfate (ZnSO_4_) electrolyte solutions. Via physicochemical and electrochemical characterizations, we demonstrate that this peculiar electrolyte formulation weakens the electric double layer repulsive force, thus, favouring dense metallic zinc deposits and regulating the charge distribution at the zinc metal|electrolyte interface. When tested in Zn||VS_2_ full coin cell configuration (with cathode mass loading of 16 mg cm^−2^), the electrolyte solution containing the lanthanum ions enables almost 1000 cycles at 1 A g^−1^ (after 5 activation cycles at 0.05 A g^−1^) with a stable discharge capacity of about 90 mAh g^−1^ and an average cell discharge voltage of ∼0.54 V.

## Abstract (web summary)

Zinc metal is a promising anode material for aqueous secondary batteries. However, the unfavourable morphologies formed on the electrode surface during cycling limit its application. Here, the authors report the tailoring of the surface morphology using a lanthanum nitrate aqueous electrolyte additive.

## Introduction

Despite the advantages of aqueous Zn-based batteries (e.g., Zn-MnO_2_, Zn-Br_2_, and Zn-Air batteries), including high safety, low cost, and nontoxicity, the sustained chemical corrosion (the corrosion caused by the side-reactions between Zn metal and aqueous electrolytes) and low reversibility of Zn electrodes encumber their practical applications^1–4^. In aqueous electrolytes, the electrodeposition of hexagonal close-packed Zn metal has a strong propensity to form hexagonal platelets, which generates porous Zn depositions with irregular morphologies^5–7^. Such a porous structure is bound to exacerbate the chemical corrosion during the repeated plating and stripping of the Zn-metal phase due to the increased exposure of Zn electrodes to the electrolytes. The loose Zn particles with irregular morphology also cause the loss of electrical contact between the deposits and substrates and further deteriorates the reversibility of Zn electrodes^4^. What is worse, the dendritic Zn particles with irregular morphology may easily pierce the separator and lead to short circuits of batteries^8^.

To induce uniform Zn deposition, several strategies have been proposed: (1) constructing artificial interface layers to restrict the Zn crystal growth and protect the Zn metal electrode from detrimental reactions with the aqueous electrolyte^9–11^; (2) using substrates with a low lattice mismatch and low affinity to lock the crystal orientation for the uniform Zn electrodeposition^8,12^; (3) increasing the driving force for the nucleation of Zn deposits to induce the uniform distribution of Zn-metal nuclei^13^. However, most of the above approaches rely on the modification of the Zn electrodes or current collectors, which decrease the overall energy density of the cells^14–16^. Besides, the long-term cycling of Zn electrodes under conditions of high depth of discharge (DOD) and/or high areal capacity of Zn remains challenging^3,4^. It is highly desired to explore new solutions to enable high effective Zn deposition without sacrificing the energy density.

The morphology of the deposited Zn directly affects the reversibility of Zn electrodes and the lifespan of Zn-based batteries^17–19^. It is acknowledged that the morphology of the deposited Zn is related to multiple factors, including the intrinsic crystal anisotropy, electrolytes, substrate chemistry and geometry^8,13,20–22^ The process of Zn electrodeposition includes the desolvation and reduction of the Zn^2+^ ions, and the following formation and growth of the nucleus on conductive substrates. The final morphology of deposited metal is related to both the structure of as-formed grain crystals, which are observed as irregular hexagonal flakes for Zn, and the interactions between them^23^. Based on the Derjaguin-Landau-Verwey-Overbeek (DLVO) theory, the interactions between Zn deposits in aqueous electrolytes are mainly related to the van der Waals (VDW) attractive force and the electrostatic repulsion due to the electric double layer (EDL) of counterions (Supplementary Figs. 1, 2 and Supplementary Notes 1, 2)^24–26^. In ZnSO_4_ electrolyte, the Zn tends to electrodeposit as scattered and loose platelets, indicating a repulsive-force-governed Zn deposition process. To induce dense and compact Zn coherent electrodeposition, we need to regulate the interactions between the Zn deposits from repulsion to attraction. As the VDW force, which depends mainly on the distance between the particles, could be considered as fixed for the Zn deposits and is difficult to be manipulated, thus the possible solution is to weaken the EDL repulsion force between the Zn deposits.

Based on the Poisson-Boltzmann (PB) model, the EDL repulsive force between the negatively-charged surfaces of Zn deposits is mainly influenced by the thickness of the EDL, which is known as the Debye length (1/*κ*)^27^. Theoretically, by reducing the Debye length, the EDL repulsion force between two charged particles could be reduced. Here, we introduce La^3+^ ions, which serve as high-valence competitive ions to decrease the Debye length^28–33^, to the aqueous ZnSO_4_ electrolytes. The electrochemical and morphology characterizations confirmed the presence of the insert La^3+^ ions weakens EDL repulsive force between the Zn deposits, changes the preferred orientation of Zn deposits, and results in dense and compact Zn coherent electrodeposition. With La^3+^-modified electrolyte, the corrosion rate (the speed of corrosion loss) of Zn electrodes is significantly relieved with the corrosion current (the current produced in an electrochemical cell while corrosion is occurring) decreased from 421.6 to 6.3 μA cm^−2^,^34^ enabling a high average Coulombic efficiency of > 99.9% for 2100 plating/stripping cycles. At a current density of 10 mA cm^−2^ and DOD_Zn_ of 80%, the Zn||Zn cell with the modified electrolyte exhibited a stable Zn deposition for ~160 h. Benefitting from the stale Zn anodes, the Zn||VS_2_ cell in La^3+^-modified electrolyte with a limited Zn supply (11.6 mAh cm^−2^) and a high-loading VS_2_ cathode (16.0 mg cm^−2^) delivers a stable discharge capacity of about 90 mAh g^−1^ and an average cell discharge voltage of ~0.54 V. The as-proposed strategy demonstrates the importance of the thickness of EDL on the electrodeposition behaviors of Zn^2+^ ions and might also be applicable for other metal anodes.

## Results

### Electrochemical characterization of zinc metal electrode with La^3+^-containing aqueous electrolyte solution

**Fig. 1:**
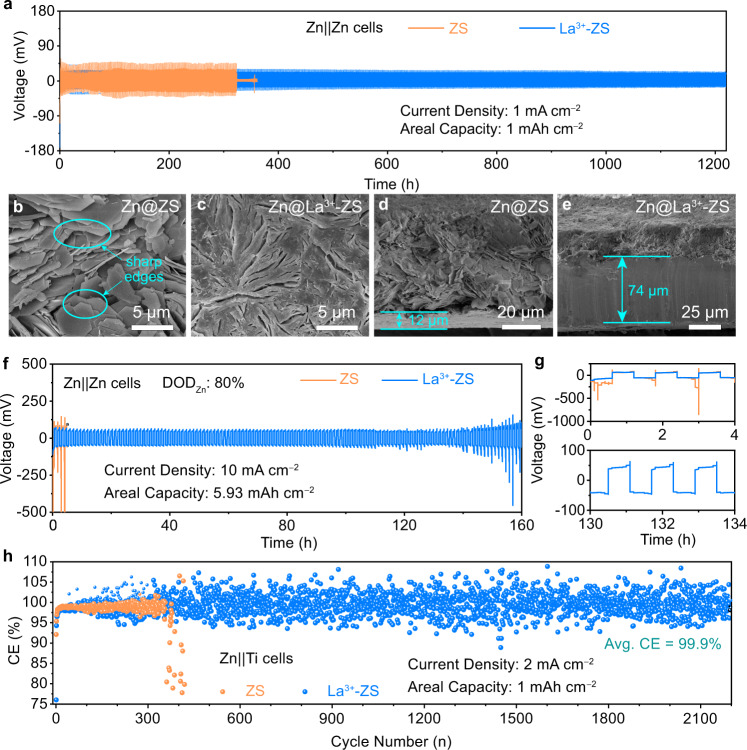
The plating/stripping behaviors for Zn electrodes in ZS and La^3+^-ZS electrolytes. **a** Cycling performance of the Zn||Zn cells with a current density of 1 mA cm^−2^ and an areal capacity of 1 mAh cm^−2^; the top and cross-sectional SEM images of the Zn electrodes for Zn||Zn cells in (**b, d**) ZS and (**c, e**) La^3+^-ZS electrolytes after 100 cycles under a current density of 1 mA cm^−2^ for 1 h. **f** Cycling performance of the Zn||Zn cells with a limited Zn supply (DOD_Zn_ = 80%), a current density of 10 mA cm^−2^, and an areal capacity of 5.93 mAh cm^−2^. **g** The selected enlarged voltage profiles of (**f**). **h** CE of the Zn||Ti cells with a current density of 2 mA cm^−2^ and a cut-off charging voltage of 0.4 V.

The La^3+^-modified electrolyte (La^3+^-ZS) was prepared by dissolving La(NO_3_)_3_ into ZnSO_4_ solution (ZS), a common electrolyte for aqueous Zn-ion batteries. To verify the effects of La^3+^ on Zn deposition, we firstly compared the plating/stripping performance of Zn||Zn cells in ZS and La^3+^-ZS electrolytes with a current density of 1 mA cm^−2^ and an areal capacity of 1 mAh cm^−2^ (Fig. 1a). The Zn||Zn cell with ZS electrolyte shows a decreasing voltage hysteresis (the voltage difference between the middle of the plating and stripping curves) during the first 50 h. However, the stabilized voltage drops suddenly after ~320 h, indicating the failure of the cell. In comparison, the Zn||Zn cell with La^3+^-ZS electrolyte exhibits stable voltage profiles > 1200 h with a negligible potential fluctuation. The voltage hysteresis remains < 100 mV during cycling. Similar results are also observed at a lower current density of 0.5 mA cm^−2^, where the Zn||Zn cell with La^3+^-ZS electrolyte is cycled for more than 1800 h, much longer than the one in ZS electrolyte (~440 h, Supplementary Fig. 3).

To figure out how the La^3+^-ZS electrolyte affects the Zn electrodeposition, we disassembled the cycled Zn||Zn cells (after 100 cycles with a current density of 1 mA cm^−2^ and an areal capacity of 1 mAh cm^−2^) and analyzed the morphology of the cycled Zn electrodes by a scanning electron microscopy (SEM). As the result shown in Fig. 1b, the cycled Zn electrode in ZS electrolyte shows a porous Zn layered structure with irregular morphology which could lead to cell failure upon prolonged cycling^5^. In addition, this structure also leads to more side reactions between the Zn electrode and electrolyte due to the exposed surface, and thus more by-products accumulation. In comparison, after being cycled under a same test condition, the Zn electrode with La^3+^-ZS electrolyte displays a dense surface with the deposited Zn particles closely connected with each other (Fig. 1c). Figure 1d displays the cross-sectional SEM image of the cycled Zn electrodes in ZS electrolyte, where the Zn electrode is depleted with 12 μm in thickness of Zn foil left, indicating the formation of the large amounts of electronically-disconnected (i.e., “dead”) Zn and by-products^35,36^. In contrast, the cycled Zn electrode in La^3+^-ZS electrolyte shows a dense Zn-deposition layer with ~74 μm Zn foil left (Fig. 1e). Considering the thickness of the fresh Zn electrode used is 80 μm, the use of La^3+^-ZS electrolyte reduces the Zn consumption by 91% compared with the one in ZS electrolyte. The SEM results demonstrate that the introduction of La(NO_3_)_3_ into the ZS electrolyte induces dense Zn electrodeposition and enhances the utilization of Zn foil.

For practical applications, the depth of discharge of the Zn electrodes (DOD_Zn_) significantly affects the cycling life and the overall specific energy of the full cell^3,4^. The plating/stripping performance of the symmetrical Zn||Zn cells under different DOD_Zn_ was tested using thin Zn electrodes (13 μm, 7.40 mAh cm^−2^) with ZS and La^3+^-ZS electrolyte. Figure 1f displays the results of the cells under a condition of DOD_Zn_ = 80% (10 mA cm^−2^, 5.93 mAh cm^−2^). Under such a high DOD_Zn_, the control Zn||Zn cell with ZS electrolyte showed a sharp voltage increase at the end of the initial stripping/plating process and failed after 3 h of cycling (Fig. 1g). When La^3+^-ZS electrolyte was employed, the cell exhibited stable voltage profiles > 140 h with a voltage hysteresis of ~100 mV. When the current density was lowered to 1 mA cm^−2^, the Zn||Zn cell with La^3+^-ZS electrolyte delivered a stable cycling life for > 450 h (Supplementary Fig. 4), much longer than that with ZS electrolyte. The performance of the symmetric cells with the La^3+^-ZS electrolyte are well-positioned when compared with the state-of-the-art literature of aqueous zinc metal lab-scale cells (see Supplementary Table 1).

We further investigated the reversibility of Zn electrodes in ZS and La^3+^-ZS electrolytes with asymmetric Zn||Ti cells by plating 1 mAh cm^−2^ of Zn onto the Ti foil at a current density of 2 mA cm^−2^ and then stripping to 0.4 V. Figure 1h shows the Coulombic efficiency (CE) of both cells. The control cell with ZS electrolyte failed after < 400 plating/stripping cycles with fluctuation of the CE noticed at the end of cycle life, indicating the poor reversibility of Zn in ZS electrolyte. While when La^3+^-ZS electrolyte was used, the cell delivered 2100 plating/stripping cycles with an average CE of 99.9%^37,38^. The flat stripping/plating voltage profiles for Zn||Ti cell with La^3+^-ZS electrolyte, and the surge of charge capacity at the 400^th^ cycle for the one with ZS electrolyte support the claim of the stability of Zn electrodes in La^3+^-ZS electrolyte (Supplementary Fig. 5). Similar results are also obtained when the Ti-foil electrodes were replaced by carbon papers (Supplementary Fig. 6). Even under current densities of 10 mA cm^−2^ and 20 mA cm^−2^, the Zn electrodes in La^3+^-ZS electrolyte also demonstrated better reversibility than those of the control cells (Supplementary Figs. 7 and 8).

### Ex situ morphological and structural analyses of the zinc metal depositions

**Fig. 2:**
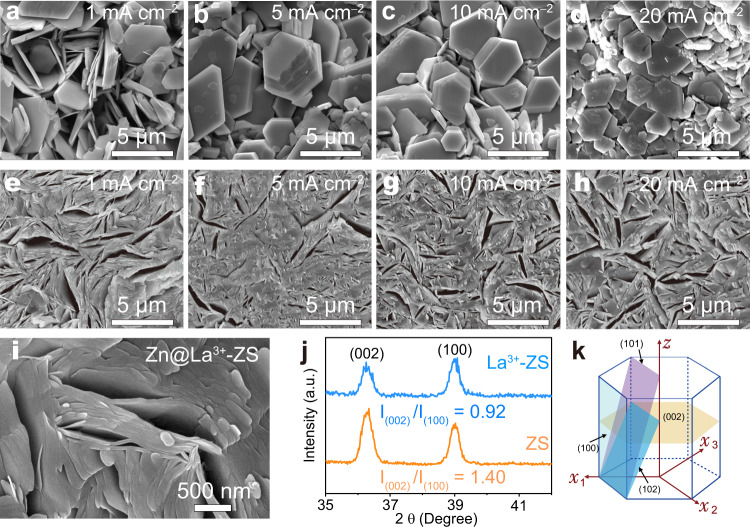
The morphology analysis of Zn deposits. The SEM images of Zn deposits from the Zn electrodes of Zn||Zn cells with a fixed areal capacity of 1 mAh cm^−2^ and different current densities from 1 to 20 mA cm^−2^ in (**a**–**d**) ZS and (**e**–**h**) La^3+^-ZS electrolytes. **i** The high-magnification SEM image of the Zn electrode for a Zn||Zn cell in La^3+^-ZS electrolyte after 10 cycles under a current density of 1 mA cm^−2^ for 1 h. **j** GIXRD patterns of Zn deposits from the Zn electrodes of a Zn||Zn cell under a current density of 1 mA cm^−2^ for 1 h showing the reduced (002) planes in La^3+^-ZS electrolyte. **k** The illustration of the hexagonal close packed (hcp) structure of Zn.

It is known that the Zn deposits in ZS electrolyte tend to exhibit a hexagonal-platelet morphology due to the lower thermodynamic free energy of the exposed (002) plane^8^. To investigate how the La^3+^-ZS electrolyte affects the electrodeposition behavior of Zn, we checked the morphology for Zn deposits in ZS and La^3+^-ZS electrolytes at different current densities with a fixed deposition amount of 1 mAh cm^−2^. As the ex situ SEM images shown in Fig. 2a–d, the Zn deposits obtained in ZS electrolyte are built with hexagonal platelets, and the thickness of the platelets increases gradually with the increase of the current densities. However, even when the current density was increased to 20 mA cm^−2^, the as-deposited platelets remained scattered. Such loose and separate structures are resulted from the strong repulsion force between the Zn deposits, which prevents the consolidation of the platelets. The Zn deposits show dense and compact morphologies in La^3+^-ZS electrolyte at all the current densities from 1 to 20 mA cm^−2^ (Fig. 2e–h). Here, the porous structures observed may origin from the nonuniform distribution of the Zn nucleation on the substrate. The high-magnification SEM image for Zn deposits at a current density of 1 mA cm^−2^ displays that the compact Zn deposits are piled with Zn platelets (Fig. 2i), the decreased presence of the porous structures between the Zn platelets reveals that the interactions between the Zn deposits have been successfully regulated from repulsion to attraction by adding La(NO_3_)_3_ into ZS electrolyte.

The structure of the Zn deposits obtained from ZS and La^3+^-ZS electrolytes was further characterized by grazing incidence X-ray diffraction (GIXRD), a powerful tool to investigate the texturing and orientation anisotropy of thin film. As the result shown in Fig. 2j, the Zn deposits obtained in La^3+^-ZS electrolyte display a relatively weaker (002) peak. Quantitatively, the relative intensity ratio of peak (002) (I_(002)_) to that of peak (100) (I_(100)_) decreases from 1.4 to 0.92, indicating that the reduced (002) planes for the Zn deposits in La^3+^-ZS electrolyte. Considering the hcp structure of Zn (Fig. 2k) and reduced (002) plane of the Zn deposits obtained from La^3+^-ZS electrolyte, it is safe to conclude that the as-obtained Zn deposits are piled up along the c axis, which could be regarded as a coherent deposition^5,39^.

### Physicochemical investigation on the role of charges for the zinc metal deposition process

**Fig. 3:**
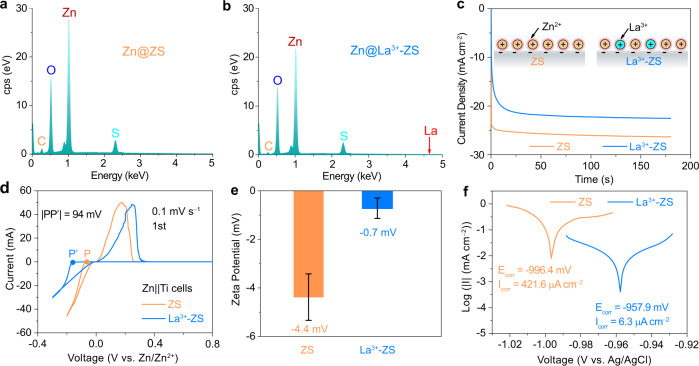
The role of the La^3+^-modified electrolyte on Zn deposition. EDS of the Zn electrodes disassembled from the Zn||Zn cells after 100 cycles at a current density of 1 mA cm^−2^ for 1 h in (**a**) ZS and (**b**) La^3+^-ZS electrolytes. **c** Chronoamperogram (CA) of the Zn electrodes of a Zn||Zn beaker cell with an Ag/AgCl reference electrode in ZS or La^3+^-ZS electrolytes at an overpotential of −200 mV. **d** Cyclic voltammograms (CV) of Zn||Ti cells in ZS and La^3+^-ZS electrolytes. **e** The statistical Zeta potentials of Zn depositions (we disassembled the Zn||Ti cell after discharging 1 h at the current density of 1 mA cm^−2^, took out the Ti electrode, and collected the deposited Zn metal on the Ti electrode.) in ZS and La^3+^-ZS electrolytes. **f** Linear polarization curves of the fresh Zn electrodes (the commercial Zn foils delivering the same surface condition and effective surface area) collected with a scanning rate of 0.1 mV s^−1^ in ZS and La^3+^-ZS electrolytes using a three-electrode cell. The three-electrode cell was constructed using the fresh Zn electrode as the working electrode, Pt wire as the counter electrode, and Ag/AgCl electrode as the reference electrode.

To distinguish the function of La(NO_3_)_3_ additive, we prepared another control electrolyte (marked as NO_3_^−^-ZS), which has the same NO_3_^−^ concentration as that of the La^3+^-ZS electrolyte (0.0255 m), by adding Zn(NO_3_)_2_ to the ZS electrolyte. The cycling stability of the Zn||Zn cells with ZS, La^3+^-ZS, and NO_3_^−^-ZS electrolytes was compared with a current density of 1 mA cm^−2^ and an areal capacity of 1 mAh cm^−2^ (Supplementary Fig. 9). The cell with NO_3_^−^-ZS electrolyte failed after ~550 cycles, longer than the control cell, but only less than half of the one using La^3+^-ZS electrolyte (> 1200 cycles). Such results imply that the improved cycling stability of the Zn electrodes in La^3+^-ZS electrolyte is mainly attributed to the La^3+^ ions. To determine how the La^3+^ ions affect the Zn plating/stripping behaviors, we further investigated the cycled Zn electrodes in ZS and La^3+^-ZS electrolytes with energy-dispersive X-ray spectroscopy (EDS). The corresponding SEM images are shown in Supplementary Fig. 10 and the selected regions of the Zn electrodes can represent the electrodes cycled in ZS and La^3+^-ZS electrolytes. As the results shown in Fig. 3a, b, only the signals belonging to C, S, O, and Zn elements are detected for both samples. The absence of the La in the cycled Zn electrode with La^3+^-ZS electrolyte suggests that the La^3+^ ions in La^3+^-ZS electrolyte are not reduced or involved in the formation of the by-products. The inert nature of La^3+^ ions guarantees the durability of the La^3+^-ZS electrolyte during long-term cycling. Here, the similar CPS amounts (in Fig. 3a, b) for Zn, O, S elements of the cycled Zn electrode only imply the similar chemical composition for the passivation layer of the cycled Zn electrodes in ZS and La^3+^-ZS electrolytes. Actually, the XRD patterns of cycled Zn electrodes confirmed the formation of the by-products is hindered in La^3+^-ZS electrolyte compared with the ones in ZS electrolyte (Supplementary Fig. 11).

The Zn deposits growth mechanism in ZS and La^3+^-ZS electrolytes was further investigated by Chronoamperometry (CA) in a three-electrode cell set-up, where the working electrode and the counter electrode both are Zn foils, and the reference electrode is an Ag/AgCl electrode (Supplementary Fig. 12). CA is an electrochemical method characterizing the concentration change of electroactive species in the vicinity of the surface^40^. The current response, which is determined by the nucleation centers^41–43^, is recorded *vs*. time of the Zn||Zn cells with ZS and La^3+^-ZS electrolytes at an overpotential of −200 mV for a deposition time of 180 s is provided in Fig. 3c. In ZS electrolyte, the current density reaches its steady value (~−26 mA cm^−2^) soon after the overpotential was applied, implying the activation of all the nucleation sites. In contrast, the current density for the cell with La^3+^-ZS electrolyte is characterized by prolonged activation time, indicating that the number of nuclei increases gradually with time and the progressive nucleation is governing during Zn deposition in La^3+^-ZS electrolyte^40,42^. It is also noticed that the steady current density in ZS electrolyte (~−26 mA cm^−2^) is higher than that in La^3+^-ZS electrolyte (~−22 mA cm^−2^). The differences in the nucleation mechanisms and steady current densities could be ascribed to the adsorption of the La^3+^ ions on the surface of the Zn electrodes, which decrease the number of the active nucleation sites and slow down the formation of the nuclei in La^3+^-ZS electrolyte.

The adsorption of metal ions on an electrode is typically regarded as a monolayer-adsorption process^44–48^. According to the Langmuir isotherm, the relationship between the equilibrium coverage (*θ*) and the concentration (*c*) of the adsorbents in the bulk solution follows Eq. (1):

1 $$\theta =\frac{{cK}}{1+{cK}}$$

Where *K* is the equilibrium constant and depends only on the electrode potential and temperature. As the concentration of La(NO_3_)_3_ in La^3+^-ZS electrolyte is only 0.0085 m, much less than that of ZnSO_4_ (2 m), the equilibrium adsorbent coverage *θ* could be regarded as the same for the Zn electrodes in ZS and La^3+^-ZS electrolytes, which means the current density (*j*) of the CA curves is proportional to the number of the adsorbed Zn^2+^ ions. In other words, the lower current density signifies less Zn^2+^ ions are adsorbed on the electrode in La^3+^-ZS electrolyte, which is the result of the completive adsorption of inert La^3+^ ions. Besides, the Zn nucleation in La^3+^-ZS electrolyte shows larger polarization voltage than that in ZS electrolyte (|PP’| = 94 mV, Fig. 3d), also reflecting the competitive adsorption between Zn^2+^ and La^3+^ ions in La^3+^-ZS electrolyte.

To analyze the net charge of the Zn deposits in ZS and La^3+^-ZS electrolytes, we disassembled the Zn||Ti cell after discharging 1 h at the current density of 1 mA cm^−2^, collected the deposited Zn metal from the Ti electrode, and checked the Zeta potentials of the deposited Zn metal. As the results shown in Fig. 3e, the deposited Zn flakes obtained from ZS electrolyte show a Zeta potential of ~−4.4 mV, indicating that the Zn deposits are negatively charged. These negatively charged Zn flakes repel with each other, leading to the porous Zn deposits in ZS electrolyte. In comparison, a Zeta potential of the Zn deposits obtained from La^3+^-ZS electrolyte is only ~−0.7 mV, implying fewer net charges on the surface of Zn flakes. The reduced net charges are attributed to that high-valence La^3+^ ions into ZS electrolyte decrease the Stern potential of Zn deposits faster than Zn^2+^ ions^30,33^, which means shorter distance is needed to reach same stern potential and Zeta potential, indicating the EDL is compressed with La^3+^ ions (Supplementary Fig. 13 and Supplementary Note 3). Fewer net charges on the surface of Zn deposits lead to a reduced EDL force between Zn deposits. Based on the DLVO theory, the interaction between the Zn deposits is determined by the EDL repulsion and the VDW attraction^23^. With the reduced EDL force between Zn deposits in La^3+^-ZS electrolyte, the VDW attraction becomes prominent, and the deposited Zn metal tend to agglomerate along the (002) plane (Supplementary Fig. 14 and Supplementary Note 4), leading to the formation of the dense and stacked Zn deposits.

The introduction of La^3+^ into ZS electrolyte also suppresses the parasitic reaction between electrolytes and Zn electrodes. As the linear polarization curves of the fresh Zn electrodes in ZS and La^3+^-ZS electrolytes shown in Fig. 3f. Compared with that of the Zn electrodes in ZS electrolyte, the corrosion potential (the characteristic or property of metal and nonmetal surfaces to lose electrons in the presence of an electrolyte) of the Zn electrode in La^3+^-ZS electrolyte increases from −996.4 to −957.9 mV (vs. Ag/AgCl)^34^, implying a lower tendency of corrosion of Zn electrodes. In addition, the corrosion current (I_corr_) of Zn electrodes is reduced from 421.6 μA cm^−2^ in ZS electrolyte to 6.3 μA cm^−2^ in La^3+^-ZS electrolyte, implying that the corrosion was inhibited by 98% with the addition of La^3+^ based on the Eq. (2)^49^.

**Fig. 4:**
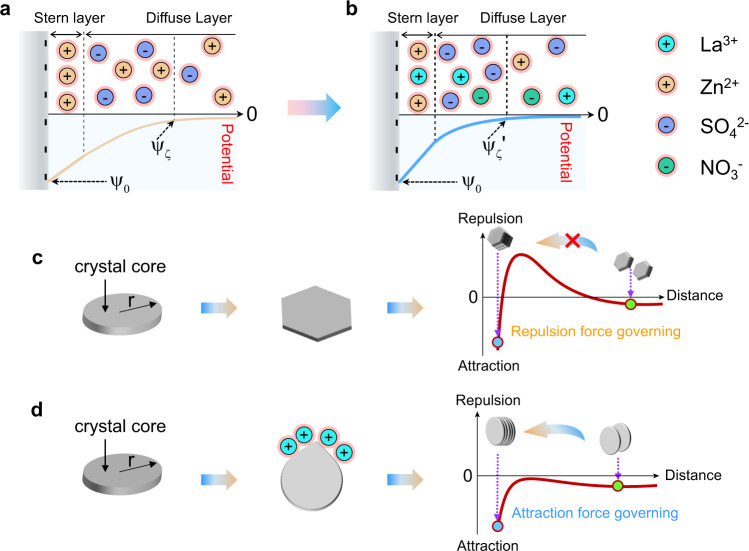
The illustrations of the coherent electrodeposition induced by a compressed electric double layer of Zn particles. The comparison of the electric double layer of the Zn deposits in (**a**) ZS and (**b**) La^3+^-ZS electrolytes; the as-proposed growth models of the Zn deposits in (**c**) ZS and (**d**) La^3+^-ZS electrolytes.

Based on the above discussion, we illustrate the schemes of Zn coherent electrodeposition induced by the compressed electric double layer of Zn deposits. As shown in Fig. 4a, b, the Zn electrode is negatively charged during electrodeposition, delivering a potential of ${{{{{{\rm{\psi }}}}}}}_{0}$. According to the electric double layer theory, positive Zn^2+^ ions are absorbed onto the Zn electrode. Thereby, the potential of the surface of Zn deposits in ZS electrolyte increases from ${{{{{{\rm{\psi }}}}}}}_{0}$ to ${{{{{{\rm{\psi }}}}}}}_{{{{{{\rm{\varsigma }}}}}}}$. While in La^3+^-ZS electrolyte, both the bivalent Zn^2+^ ions and trivalent La^3+^ ions are absorbed on the surface of Zn deposits, resulting in fewer net negative charges than that in ZS electrolyte. In this context, the surface of Zn deposits presents a higher potential ${{{{{{\rm{\psi }}}}}}}_{{{{{{\rm{\varsigma }}}}}}}{\prime}$ than ${{{{{{\rm{\psi }}}}}}}_{{{{{{\rm{\varsigma }}}}}}}$. The EDL repulsion decreases due to the fewer net charges, and the electric double layer gets thinner in a La^3+^-ZS electrolyte than in a ZS electrolyte^50^. As illustrated in Fig. 4c, in ZS electrolyte, the electrodeposited Zn tends to grow into separate hexagonal plates due to the EDL repulsion dominated interactions between the Zn deposits^32,51^. While when La^3+^-ZS electrolyte is used, the competitive adsorption of the inert La^3+^ ions on the surface of the Zn electrodes reduces the EDL repulsion between the Zn deposits and leads to the coherent electrodeposition of the Zn deposits along (002) plane (Fig. 4d).

### Electrochemical energy storage testing in Zn||VS_2_ coin cell configuration

**Fig. 5:**
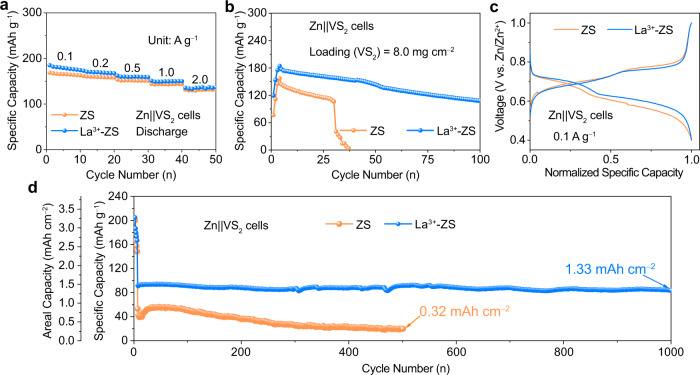
The electrochemical performance of Zn||VS_2_ cells in ZS and La^3+^-ZS electrolytes. **a** The rate performance. **b** The cycling performance with a limited Zn supply (N/P capacity ratio: 4.3). **c** Normalized charge-discharge curves of (**b**) at the 20^th^ cycle. **d** The cycling performance with a limited Zn supply (11.6 mAh cm^−2^) and a high-loading VS_2_ cathode (16.0 mg cm^−2^) at a current density of 16.0 mA cm^−2^.

The La^3+^-ZS electrolyte was also tested in full coin cell configuration using a Zn metal anode and a VS_2_-based cathode (Fig. 5). Here, the VS_2_ was synthesized via a hydrothermal method (Supplementary Fig. 15). The Zn||VS_2_ cell with La^3+^-ZS electrolyte exhibits slightly higher specific capacities than those with ZS electrolyte at specific current of 0.1, 0.2, 0.5, 1.0, and 2.0 A g^−1^ (Fig. 5a). The higher and more stable CE values during cycling indicate that La^3+^-ZS electrolyte benefits the Zn||VS_2_ cell performance (Supplementary Figs. 16). We further tested the cycling performance of the Zn||VS_2_ cell with a limited Zn supply (7.4 mAh cm^−2^) and a high-loading cathode (8.0 mg cm^−2^) at a specific current of 0.1 A g^−1^ after 5 activation cycles at 0.05 A g^−1^. As the results displayed in Fig. 5b, the Zn||VS_2_ cell with ZS electrolyte shows a rapid capacity decay and failed after 30 cycles. Whereas the cell with La^3+^-ZS electrolyte remains a discharge capacity of 108 mAh g^−1^ after 100 cycles. The normalized discharge/charge profiles of the Zn||VS_2_ cell at the 20^th^ cycle were compared in Fig. 5c, where the cell in ZS electrolyte shows larger voltage hysteresis (the voltage gap at 50% capacity) than that of the one in La^3+^-ZS electrolyte. In addition, the voltage hysteresis of the cells in La^3+^-ZS electrolyte decreases slightly from 118 mV in the 6^th^ cycle to 101 mV in the 24^th^ cycle, whereas the ones in ZS electrolyte increases gradually with cycles (Fig. 5c and Supplementary Fig. 17). The long-term cycling stability of Zn||VS_2_ cells in ZS and La^3+^-ZS electrolytes at a specific current of 1 A g^−1^ are compared in Supplementary Fig. 18. Benefitting from the improved stability and reversibility of the Zn electrode in La^3+^-ZS electrolyte, the Zn||VS_2_ cell with La^3+^-ZS electrolyte delivers cycling stability (1000 cycles) with a high average CE of 99.89%. In comparison, the Zn||VS_2_ cell with ZS electrolyte faded quickly and failed in less than 400 cycles. Furthermore, compared with that of the one using ZS electrolyte, the cycling performance of the Zn||VS_2_ cell with a limited Zn supply (11.6 mAh cm^−2^) and a high-loading VS_2_ cathode (16.0 mg cm^−2^) using La^3+^-ZS electrolyte showed significantly improved cycling stability and higher average discharge voltage (the voltages of cells at medium discharge capacity) 0.54 V vs. 0.49 V at a high areal current density of 16.0 mA cm^−2^ during the long-term cycling (Fig. 5d and Supplementary Fig. 19), which benefit from the intact Zn electrodes (Supplementary Fig. 20).

In summary, by introducing high-valence La^3+^ ions into aqueous ZnSO_4_ electrolyte, we successfully compressed the EDL, reduced the EDL repulsion between the Zn deposits, and obtained coherent-electrodeposited Zinc with a compact structure and improved electrochemical stability. With this EDL-compressing approach, a stable Zn plating/stripping performance for > 1200 h, a high average Coulombic efficiency of 99.9% for over 2100 cycles, a prolonged cycling stability under a deep-discharge condition (80% DOD_Zn_), and stable Zn||VS_2_ coin cell performance were realized.

## Methods

### The preparation of the electrolytes

The ZS electrolyte (2 m ZnSO_4_) was prepared by dissolving 17.25 g of ZnSO_4_ (99.995%, Aladdin) into 30 ml of deionized (DI) water. The La^3+^-ZS electrolyte (2 m ZnSO_4_ and 0.0085 m La(NO_3_)_3_) was prepared by adding 110.4 mg of La(NO_3_)_3_ (99%, Aladdin) into 30 mL of ZS solution. To inquire the role of NO_3_^−^, NO_3_^−^-ZS electrolyte with a same concentration of NO_3_^−^ as La^3+^-ZS was prepared by adding 113.8 mg of Zn(NO_3_)_2_ (AR, Sinopharm Chemical) into 30 mL of ZS solution.

### Synthesis of VS_2_ powders

VS_2_ Powders were synthesized by a hydrothermal method^52^. In a typical procedure, 3 ml of NH_3_H_2_O and 23 mmol of thioacetamide (TAA) was added to a 45 ml of 3 mmol NH_4_VO_3_ solution one by one with an interval time of 1 h under stirring. After being stirred for another hour, the as-received brown mixture was transferred into a 50 ml Teflon-lined autoclave and maintained at 180 °C for 20 h. After the reaction, the solid product was collected by centrifuging and washing with DI water and ethanol thoroughly and dried in a vacuum oven at 50 °C overnight. Finally, the as-collected black powders were annealed in N_2_ at 180 °C for 8 h to obtain the VS_2_ powders.

### Characterizations

The morphologies and structures of the samples were characterized by field emission scanning electron microscopy (SEM, Nova NanoSEM 450) equipped with energy-dispersive X-ray spectroscopy (EDS). X-ray diffraction (XRD) patterns were recorded with a Bruker-AXS microdiffractometer (D-8 ADVANCE) using Cu-K_α1_ radiation (λ = 1.5406 Å) from 10° to 90°. Grazing incidence X-ray diffraction (GIXRD) patterns were collected from 35 to 47° on a Rigaku SmartLab X-ray diffractometer with a Cu-K_α1_ radiation with a step size of 0.0001°. All the electrode samples were collected by disassembling the Zn||Zn cells after a certain number of cycles at different currents. After being rinsed in DI water and dried in the air, the sampled electrodes were transferred into the SEM and XRD equipment for the morphology analysis of Zn deposits.

### Electrochemical measurements

All the electrochemical measurements are conducted in air, and the temperature is controlled at 25.0 ± 2.0 °C. The VS_2_ electrodes were prepared by a blade-cast method. Briefly, the VS_2_ powders, acetylene black carbon, and polyvinylidene fluoride (PVDF) were mixed with a weight ratio of 7: 2: 1 in N-methyl-2-pyrrolidone (NMP) with an electric mixer (AR-100, THINKY). Then, the as-prepared slurry was blade-casted onto a Ti foil (15 µm in thickness, 99%, Dongguan XingYe Metal Material). After being dried in a vacuum oven at 50 °C overnight, VS_2_ electrodes were obtained by cutting the above Ti foil into circular sheets with a diameter of 8 mm. The CR-2032 coin cells were assembled using glass fiber (GF-B, Φ19) as separators, Zn plates or VS_2_ electrodes as the working electrodes, and Zn plates (80 µm in thickness unless otherwise specified, 99%, Dongguan XingYe Metal Material), Ti foils, or carbon papers (90 µm in thickness, 98.5%, CeTech) as the counter electrodes, 100 μl of electrolytes were added to each cells. For the electrochemical characterizations of the Zn||VS_2_ cells with a limited Zn supply (13 μm, 7.40 mAh cm^−2^), the areal mass loading of VS_2_ electrodes was ~8.0 mg cm^−2^, while the number is ~1.0 mg cm^−2^ for all other Zn||VS_2_ cells. The CE tests was measured with Zn||Ti (or Zn||C) cells with a Zn deposition of 1.0 mAh cm^−2^ (2 mA cm^−2^ for 0.5 h) and a charge cut-off voltage of 0.4 V. All the above tests were performed on a Neware Battery Tester. To test the corrosion rate of Zn foil, a three-electrode cell was constructed using Zn foil as the working electrode, Pt wire (Φ0.5, 99.99%, Gaoss Union) as the counter electrode, and Ag/AgCl electrode as the reference electrode and tested on an electrochemical workstation (VMP3, Bio-Logic). The efficiency of protection (*η*%) for Zn electrodes in a La^3+^-ZS electrolyte was calculated by using the values of the corrosion current I_corr_ shown as the Eq. 2:

2 $${\eta } \% =\left(\frac{{{{{{{\rm{I}}}}}}}_{{{{{{\rm{corr}}}}}}\left({{{{{\rm{ZS}}}}}}\right)}-{{{{{{\rm{I}}}}}}}_{{{{{{\rm{corr}}}}}}\left({{{{{\rm{{La}}}^{3+}-ZS}}}}\right)}}{{{{{{{\rm{I}}}}}}}_{{{{{{\rm{corr}}}}}}\left({{{{{\rm{ZS}}}}}}\right)}}\right)\times 100$$

The linear polarization curve, chronoamperometry (CA) (at overpotentials of −200 mV), and cyclic voltammetry (CV) curves at a scan rate of 0.1 mV s^−1^ were recorded electrochemical workstation (VMP3, Bio-Logic). The Zeta potential was collected on a Zeta potential analyzer (Malvern Zetasizer Nano ZS90).

## Supplementary information

Supplementary information

## Data Availability

All data that support the findings of this study are available from the corresponding author on reasonable request. Source data are provided with this paper.

## Source data

Source Data
