# Supplementary material for: Lanthanum nitrate as aqueous electrolyte additive for favourable zinc metal electrodeposition
Source: Nat Commun. 2022 Jun 6;13:3252. doi: 10.1038/s41467-022-30939-8 (PMC9170708; doi:10.1038/s41467-022-30939-8)
Supplement: Supplementary file 1 — Supplementary information [file 41467_2022_30939_MOESM1_ESM.pdf]

Supplementary Information

Lanthanum Nitrate as Aqueous Electrolyte Additive for Favourable Zinc Metal Electrodeposition

Ruirui Zhao[1], Haifeng Wang[1], Haoran Du[1], Ying Yang[1], Zhonghui Gao[1], Long Qie[1, 2*], and Yunhui Huang[2*]

[1] Institute of New Energy for Vehicles, School of Materials Science and Engineering, Tongji University, Shanghai, 201804, China. [2] State Key Laboratory of Material Processing and Die & Mold Technology, School of Materials Science and Engineering, Huazhong University of Science and Technology, Wuhan, Hubei Province, 430074, China.
*Corresponding author.
E-mail: qie@hust.edu.cn; huangyh@hust.edu.cn

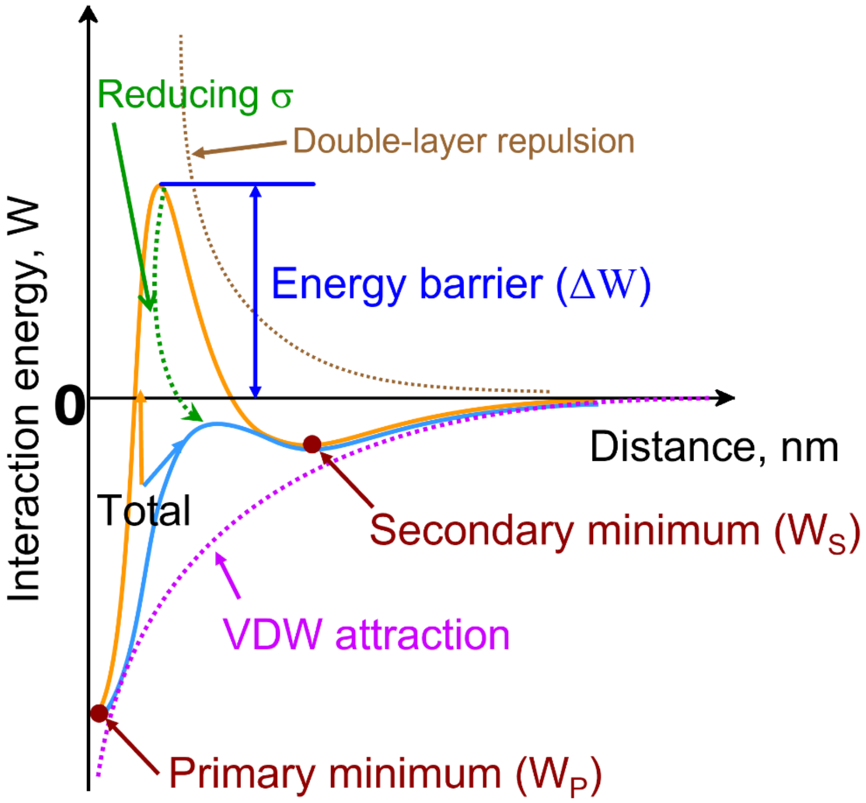

Supplementary Figure 1 The schematic of the interaction energy-distance curve described by DLVO theory[1].

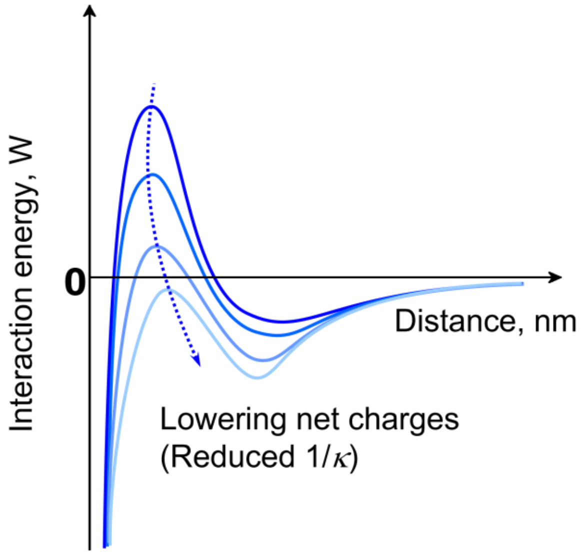

Supplementary Figure 2 The evolution of the total interaction energy with different net charges of the particles[1].

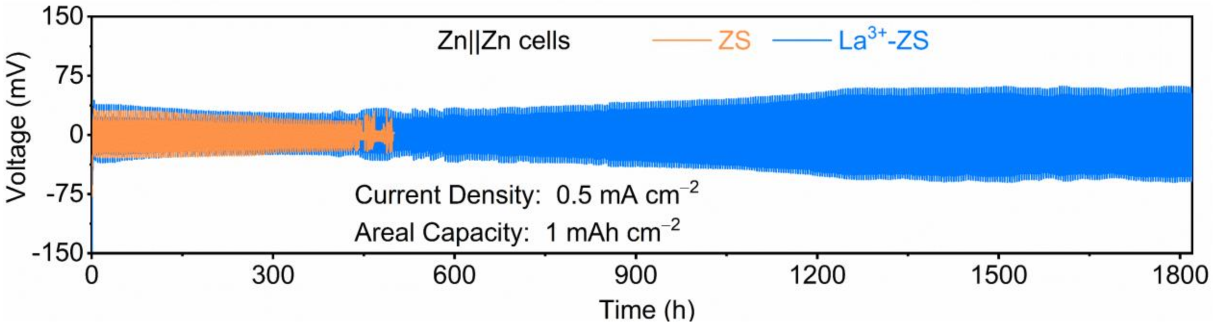

Supplementary Figure 3 The plating/stripping performance for Zn electrodes in Zn||Zn symmetrical cells with ZS and La^{3+}-ZS electrolytes under a current density of 0.5 mA cm^{-2} for 2 h.

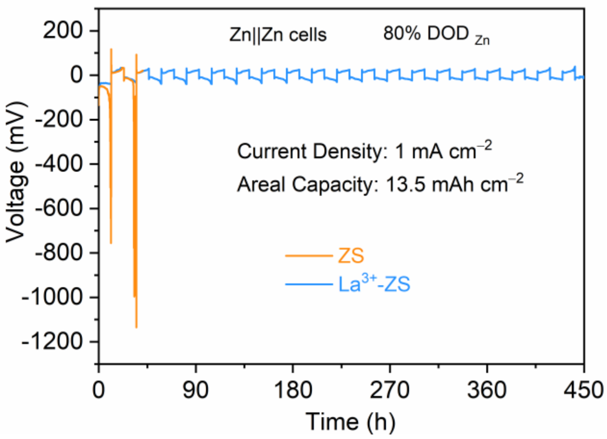

Supplementary Figure 4 The cycling performance of the Zn||Zn cell with a limited Zn supply (DOD_{Zn} = 80%) at a current density of 1 mA cm^{-2} and an areal capacity of 13.5 mAh cm^{-2}.

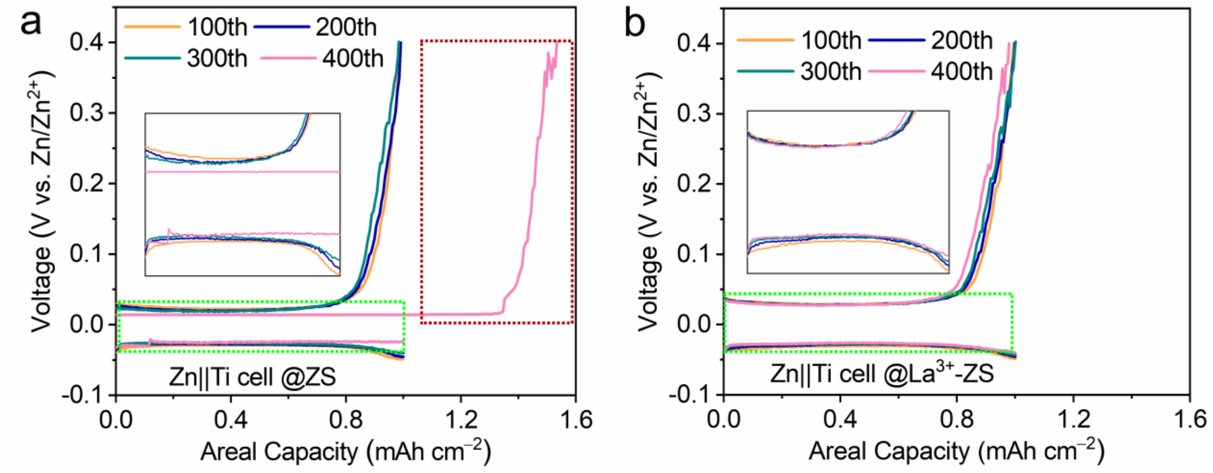

Supplementary Figure 5 The charge/discharge curves of the Zn||Ti cells with (a) ZS and (b) La^{3+}-ZS electrolytes, where discharging under a current density of 1 mA cm^{-2} for 1 h and charging under the same current density to 0.4 V.

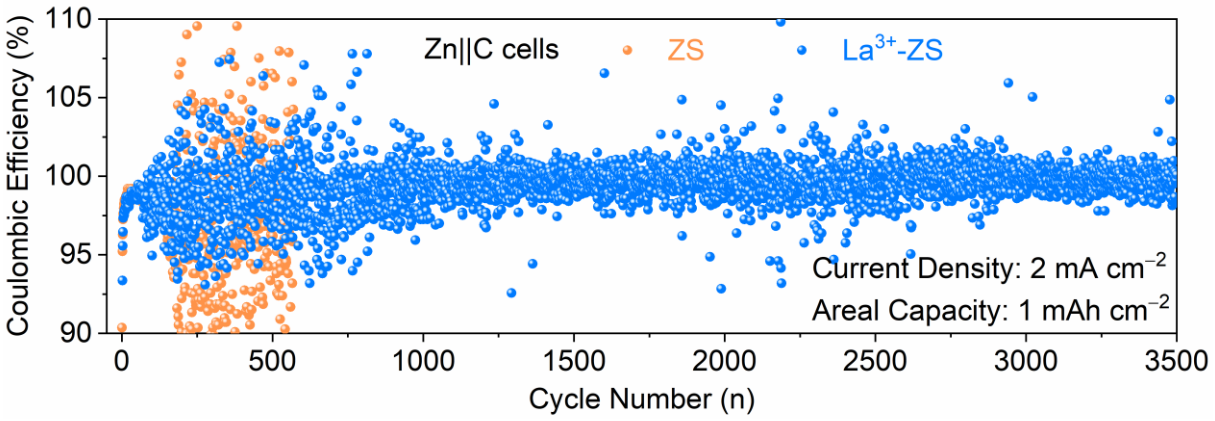

Supplementary Figure 6 The CE of Zn||C cells using ZS and La^{3+}-ZS electrolytes with a current density of 2 mA cm^{-2} and an areal capacity of 1 mAh cm^{-2}.

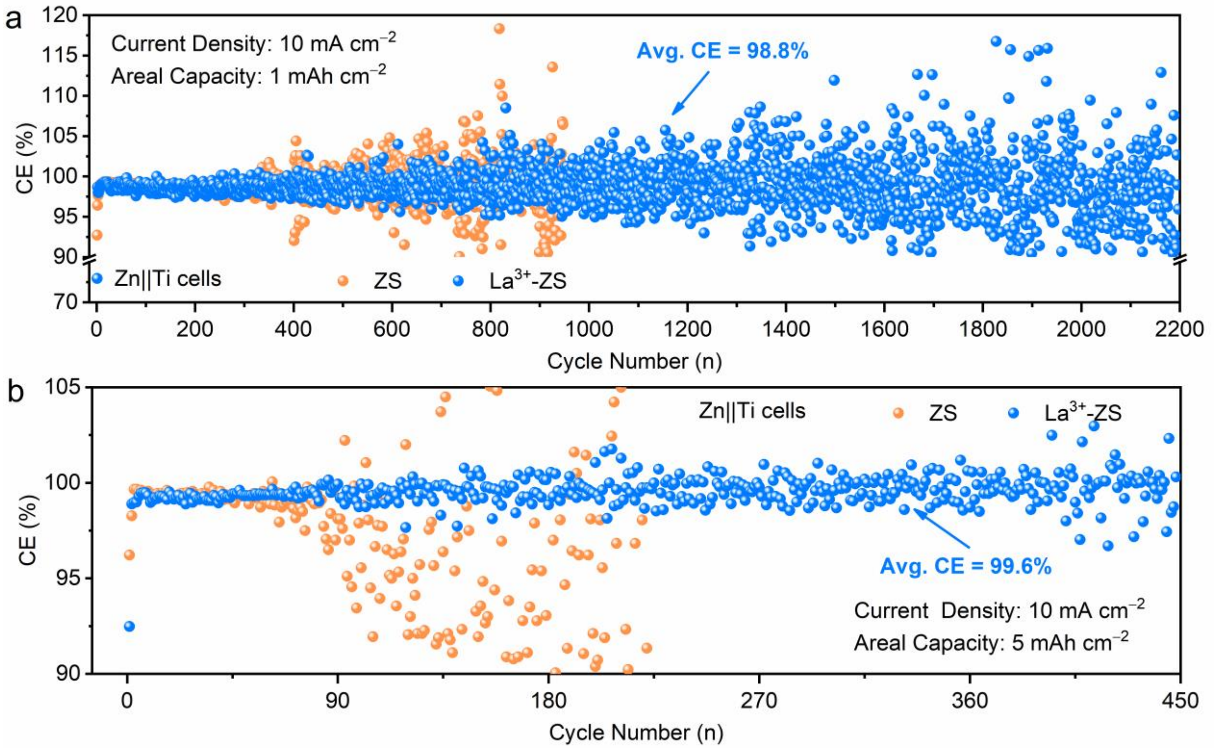

Supplementary Figure 7 CE of the Zn||Ti cells with a current density of 10 mA cm^{-2} and a cut-off stripping voltage of 0.4 V , and areal capacities of (a) 1 mAh cm^{-2} and (b) 5 mAh cm^{-2}.

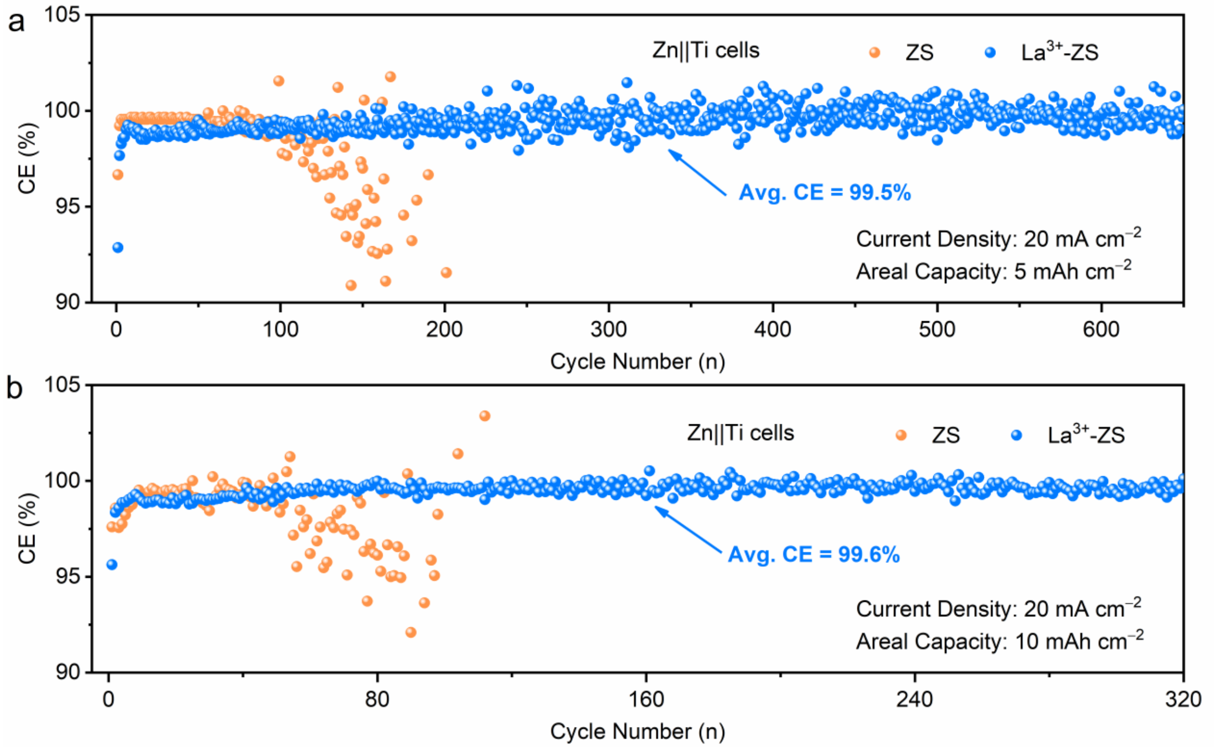

Supplementary Figure 8 CE of the Zn||Ti cells with a current density of 20 mA cm^{-2}, a cut-off stripping voltage of 0.4 V, and areal capacities of (a) 5 mAh cm^{-2} and (b) 10 mAh cm^{-2}.

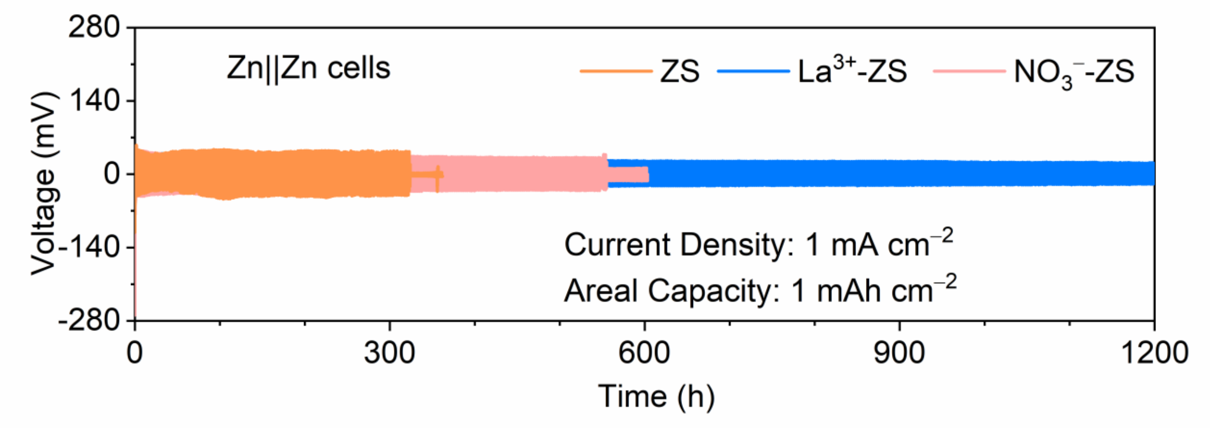

Supplementary Figure 9 The Zn plating/stripping performance of Zn||Zn cells in ZS, La^{3+}-ZS, and NO_3^--ZS electrolytes.

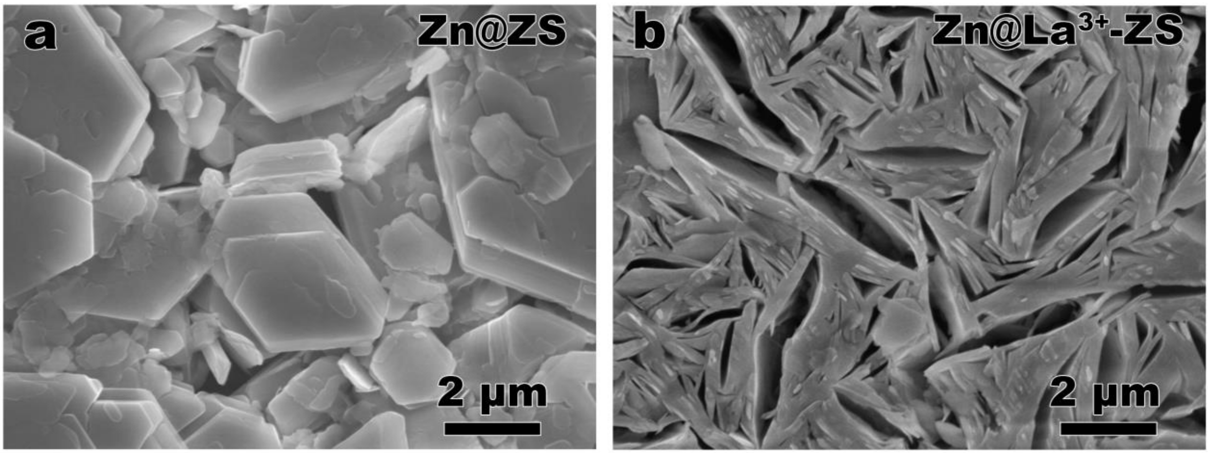

Supplementary Figure 10 The selected area for the EDS measurement of Zn electrodes after 100 cycles under a current density of 1 mA cm^{-2} for 1 h in (a) ZS and (b) La^{3+}-ZS electrolytes.

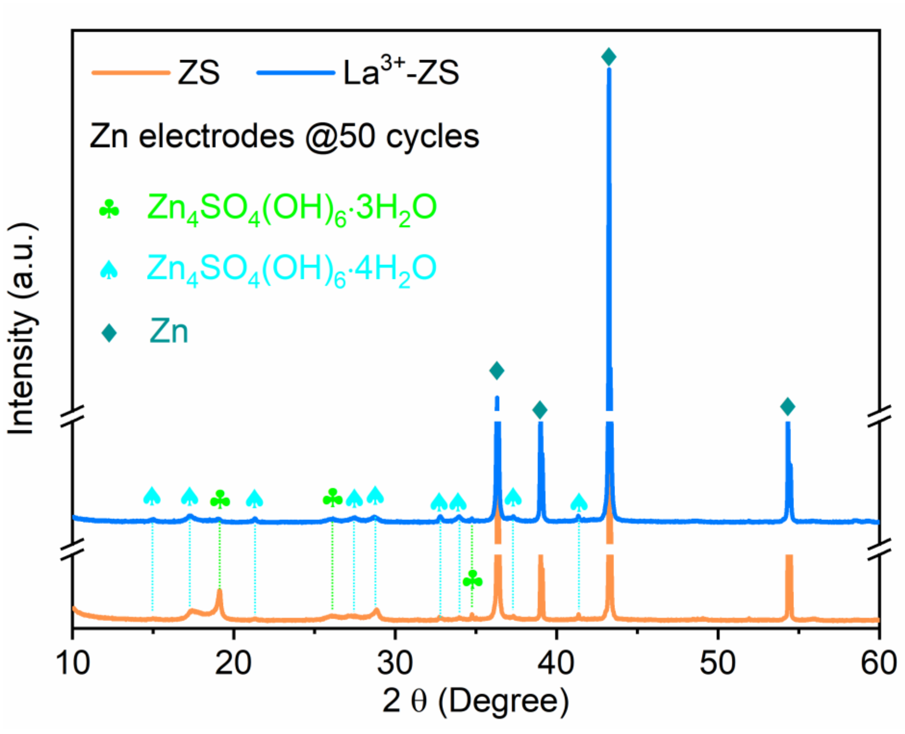

Supplementary Figure 11 XRD patterns of Zn electrodes in Zn||Zn cells after 50 cycles under a current density of 1 mA cm^{-2} for 1 h with ZS and La^{3+}-ZS electrolytes.

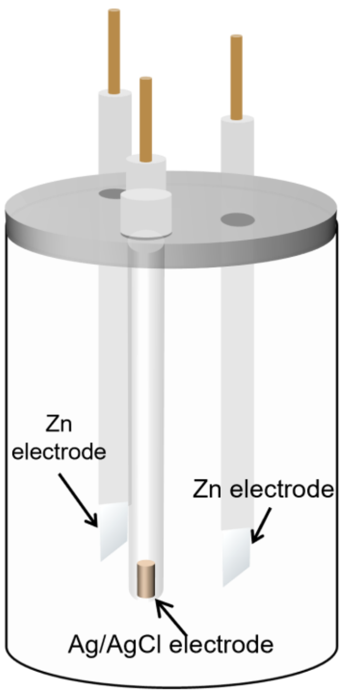

Supplementary Figure 12 Schematic of the three-electrode cell used for the CA measurements.

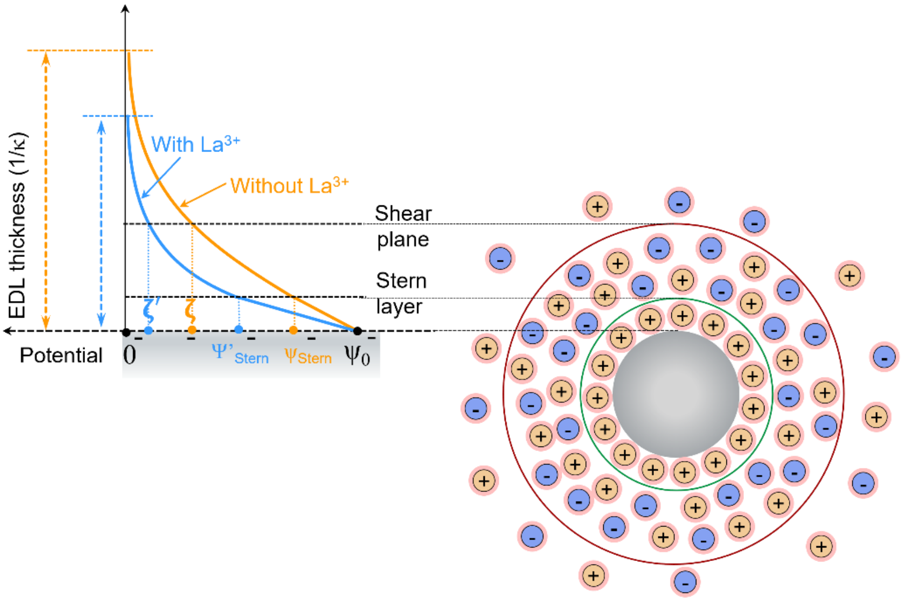

Supplementary Figure 13 The illustrations of La^{3+} ions on decreasing the Debye length ($1/\kappa$) and compressing the electric double layer of Zn deposits[2].

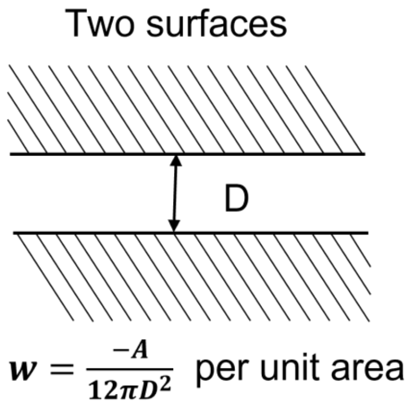

Supplementary Figure 14 Van der Waals interaction free energies for two planar surfaces based on the Hamaker summation method[3].

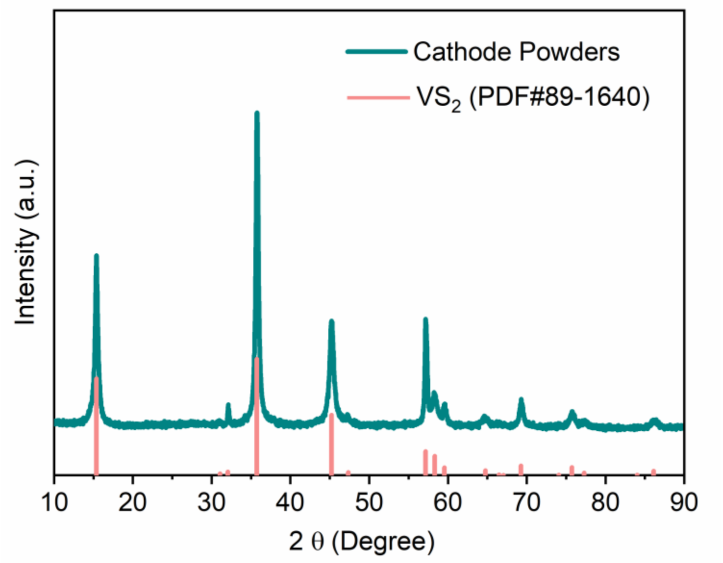

Supplementary Figure 15 The XRD pattern of the VS_2 powder.

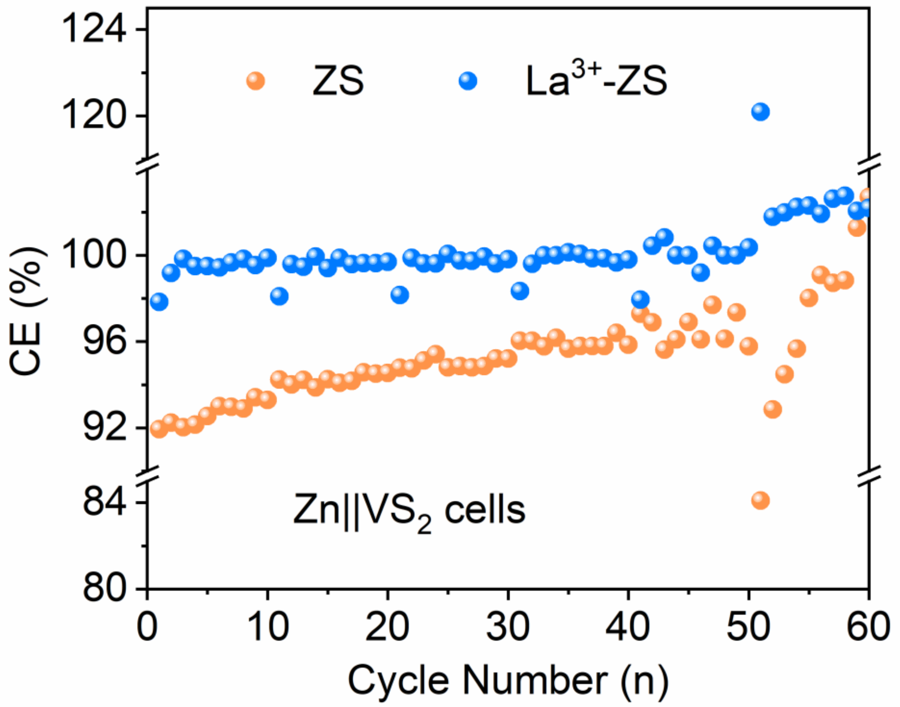

Supplementary Figure 16 The CE plots of the cycles disclosed in Figure 5a for Zn||VS_2 cells in ZS and La^{3+}-ZS electrolytes at specific current of 0.1, 0.2, 0.5, 1.0, and 2.0 A g^{-1}.

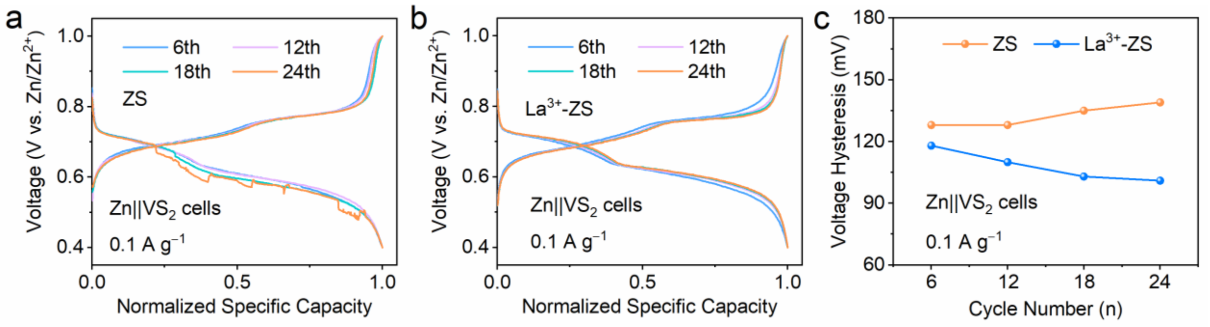

Supplementary Figure 17 The normalized charge-discharge curves of Zn||VS_2 cells with a limited Zn supply in (a) ZS and (b) La^{3+}-ZS electrolytes at different cycles under a specific current of 0.1 A g^{-1}, and (c) the comparisons of voltage hysteresis of Zn||VS_2 cells at medium discharge-capacity in both electrolytes.

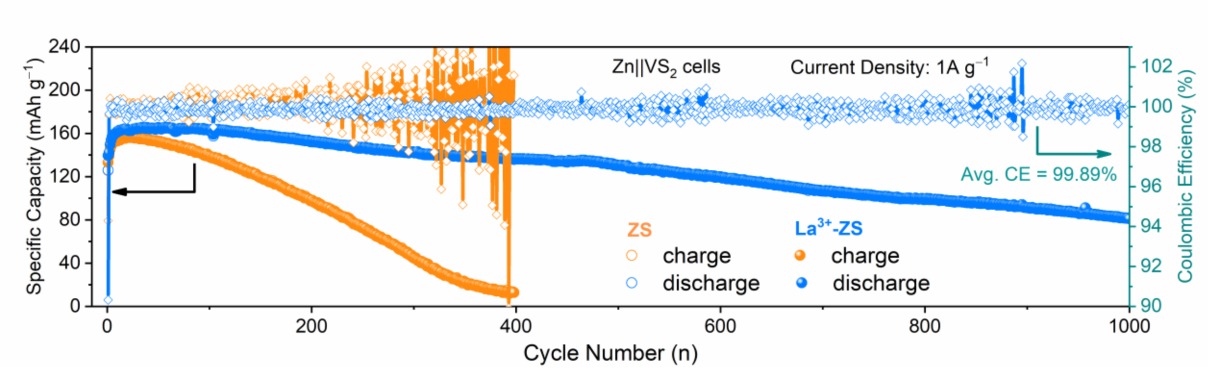

Supplementary Figure 18 The cycling performance of Zn||VS_2 cells in ZS and La^{3+}-ZS electrolytes at a current density of 1.0 A g^{-1} after 5 activation cycles at 0.05 A g^{-1}.

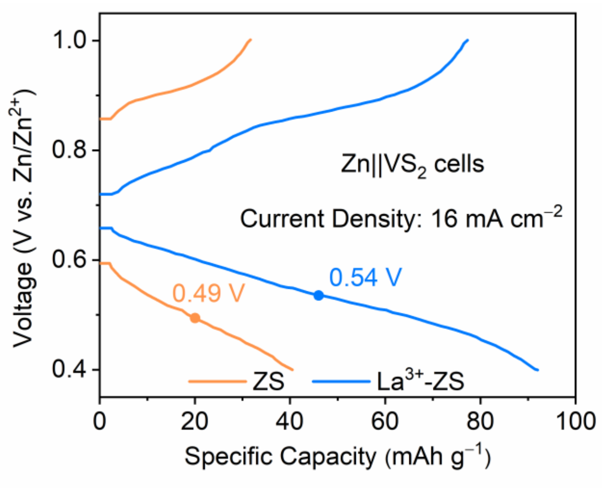

Supplementary Figure 19 The charge-discharge curves of Zn||VS_2 cells with a limited Zn supply in ZS and La^{3+}-ZS electrolytes at the 6th cycle under a current density of 16 mA cm^{-2}.

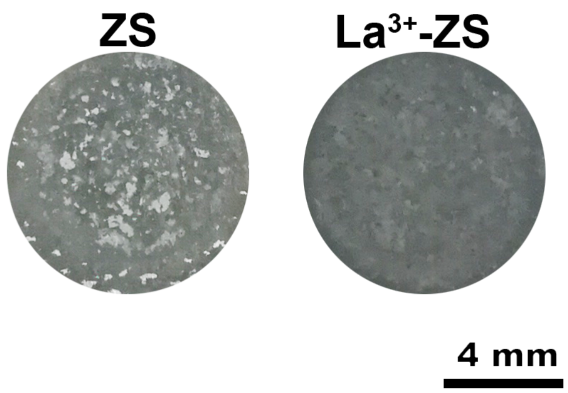

Supplementary Figure 20 The photographic pictures of cycled Zn electrodes disassembled from the Zn||VS_2 cells after 1000 cycles under a specific current of 1.0 A g^{-1} with ZS and La^{3+}-ZS electrolytes.

Supplementary Table 1 Comparisons of the test conditions and electrochemical performance of our results with the previously reported ones.

Cell Type	Electrolyte	Electrolyte dosage (μL)	Current Density (mA cm^{-2})	Cycling Life (h)	DOD_{Zn} (%)	Areal Capacity (mAh cm^{-2})	Method	Ref.
Zn‖Zn	2 m $ZnSO_4$ + 8.5 m $La(NO_3)_3$	100	10	160	80	5.93	Electrolyte optimization	This work
			1	450	80	13.5		
Zn‖Zn	2 M $ZnSO_4$ + 0.5 g L^{-1} saccharin	~	10	550	22.2	10	Electrolyte optimization	4
Zn‖Zn	1 M $ZnSO_4$ + 10 mM glucose	75	5	270	8.5	5	Electrolyte optimization	5
Zn‖Zn	1 M $ZnSO_4$ + 0.01 M $Ce_2(SO_4)_3$	~	1	400	5.7	1	Electrolyte optimization	6
Polyimide@Zn‖ Polyimide@Zn	2 M $ZnSO_4$	~	4	800	80	0.6	Surface modifications	7
Zn-CNT‖Zn-CNT	2 M $ZnSO_4$	200	5	110	35	2.5	Zn/CNT anode	8
Polyamide@Zn‖ Polyamide@Zn	2 M $ZnSO_4$	~	10	75	85	10	Surface modifications	9
Zn@ZnF_2‖ Zn@ZnF_2	2 M $ZnSO_4$	~	10	590	8.5	10	Surface modifications	10
Zn@ZnO‖ Zn@ZnO	2 M $ZnSO_4$	~	5	500	1.3	1.25	Surface modifications	11
Ionic-liquid-gel@Zn‖Ionic-liquid-gel@Zn	2 m $ZnSO_4$	~	0.5	400	90	1.8	Surface modifications	12
Zn-Mn alloy‖Zn-Mn alloy	2 M $ZnSO_4$	~	80	800	10.9	16	Zn-Mn alloy anodes	13
Zn@Nafion-Zn-X‖Zn@Nafion-Zn-X	2 M $ZnSO_4$	80	1	1000	56.9	10	Surface modifications	14
TiO_2-PVDF@Zn‖TiO_2-PVDF@Zn	1 M $ZnSO_4$	~	2	280	11.4	2	Surface modifications	15

Supplementary Note 1

It has been realized long ago that the forces acting between solid surfaces play a critical role in the interactions of nanoparticles in solutions[16]. Derjaguin, Landau, Verwey, and Overbeek (DLVO) recognized that the interactions could be approximated by the superposition of the attractive van der Waals (VDW) forces and repulsive electric double layer (EDL) forces acting between the particles involved[17-18]. The DLVO theory was first used to explain the tendency of colloids to agglomerate or remain discrete in aqueous electrolytes solutions, and further used to study the behaviors of particle deposition to water-solid interfaces[19].

As shown in **Supplementary Figure 1**, the total interaction energy of solid particles in aqueous solutions could be described as the sum of the VDW attraction and the EDL repulsion[20]. When two particles approach each other, both the VDW attraction ($W_{VDW} \propto -1/D^n$) and the EDL repulsion increase as they get closer. At each distance (D), the net interaction energy is calculated by subtracting the smaller value is from the larger value. The net value is then plotted, above if repulsive and below if attractive. At very close separation distances, the total interaction energy results in a deep attractive, which is the primary minimum (W_P). At relatively larger separation distances, the net energy curve goes through a maximum (energy barrier, ΔW) and then passes through a shallow minimum (secondary minimum, W_s) as the VDW attraction decreases slower with the distance increases than that of the EDL repulsion. At secondary minimum, particles form weak attractions but are easily redispersed, and the adhesion is reversible. While at the primary minimum, VDW attractive overpowers the EDL repulsion at low molecular distances, and the particle agglomeration is not reversible.

For the electrochemical Zn deposits in traditionary $ZnSO_4$ electrolyte, the maximum energy barrier ΔW from the secondary minimum to the primary minimum is too high to overcome, thus causing the deposited Zn a loose morphology. In this context, to induce dense and compact Zn deposition, we need to lower the energy barrier.

Supplementary Note 2

The energy barrier ΔW could be reduced by lowering the net charges of particles (**Supplementary Figure 2**). Based on the Poisson-Boltzmann (PB) model, the EDL repulsive force between the charged particles is mainly influenced by the thickness of the EDL, which is a measure of a charge carrier's net electrostatic effect in a solution and known as the Debye length ($1/\kappa$)[21]. Theoretically, by reducing the EDL thickness or the Debye length ($1/\kappa$), the EDL repulsion force between two charged particles could be reduced[22], thus inducing dense Zn deposition. With this theory foundation, for the negative-charged Zn deposits, we choose high-valence La^{3+} ions, as competitive ions to decrease the net charge and Debye length, and thus decrease energy barrier and regulate the distance between the Zn deposits into the range of the primary minimum (dense Zn deposition).

Supplementary Note 3

In $ZnSO_4$ (ZS) electrolyte, the Zn electrode is negatively-charged during the Zn^{2+} electrodeposition process, the first layer (Stern layer) formed on the electrode consists of the adsorbed Zn^{2+} ions, while the second layer (diffuse layer) is mainly composed of anions (such as SO_4^{2-}) those attracted to the Zn^{2+} ions in the first layer via the Coulomb force. When high-valence La^{3+} ions are introduced into the $ZnSO_4$ electrolyte, fewer cations (including both La^{3+} and Zn^{2+}) are needed to neutralize the negatively-charged Zn electrode in the stern layer, which could be regarded as the reduced EDL thickness.

Actually, the influencing factors on the thickness of EDL has been well studied[23]. In theory, Debye length (κ^{-1}) is used to characterize the length or thickness of the diffuse electric double layer for charged particles. In a system of N different species of charges, the j-th species carries charge q_j and has concentration n_j, Debye length could be mathematically described by Debye-Hückel equation as[24]:

$$\kappa^{-1} = \sqrt{\frac{\varepsilon \kappa_B T}{\Sigma_{j=1}^{N} n_j^0 q_j^2}} \tag{1}$$

Where, ε, κ_B, and T are, respectively, the permittivity, temperature, and Boltzmann constant. Base on Equation S1, when La^{3+} ions are introduced into the $ZnSO_4$ electrolyte, the total ionic strength ($I \propto \Sigma_{j=1}^{N} n_j^0 q_j^2$) increases, and thus leading to a reduced Debye length (κ^{-1}) and a compressed electric double layer of Zn deposits.

The reduced EDL could also be explained from the view of surface potential. As shown in **Supplementary Figure 13**, with the introduction of high-valence La^{3+} ions into ZS electrolyte, the Stern potential (ψ_{Stern}) of the Zn deposits decreases (blue line) faster than that of the ones with ZS electrolyte (orange line), which means a shorter distance is needed to reach the same stern potential and Zeta potential (ζ), indicating the EDL is compressed with La^{3+} ions. It has also been previously reported that a small amount of high-valence cations could dramatically influence the Zeta potential of charged surfaces[25-26] and La^{3+} ions have been used to induce the aggregation of liposomes by eliminating the repulsive barrier[27-28].

The decreased EDL thickness was also confirmed by the experimental results. Zeta potential, the potential of the shear plane versus the potential of the bulk electrolyte, could be used to characterize the thickness of the EDL. We measured the Zeta potentials of Zn flakes in both ZS and La^{3+}-ZS electrolytes. The results in **Figure 3e** show that Zeta potential (ζ') on the surface of Zn flakes in La^{3+}-mortified electrolyte is significantly lower than that in $ZnSO_4$ electrolyte (ζ), which could be explained as the EDL of Zn deposits is compressed with the $La(NO_3)_3$ additive[27-28].

Supplementary Note 4

In this work, the VDW free energy for Zn deposits is calculated according to the interaction forces between two surfaces (**Supplementary Figure 14**). The Hamaker constant A is defined as the Supplementary Equation (2)[3]:

$$A = \pi^2 C \rho_1 \rho_2 \quad (2)$$

where ρ_1 and ρ_2 are the number of atoms per unit volume for the surfaces, and C is the coefficient in the atom-atom pair potential. As a result, VDW attraction is largest when Zn deposits stack along the (002) plane of the closest packed plane crystal structure.

References List

1. N. Israelachvili, J. Intermolecular and Surface Forces. Ch. 12, Page 248, (Harcourt Brace & Company, 1991).
2. Yukselen, Y. & Kaya, A. Zeta potential of kaolinite in the presence of alkali, alkaline earth and hydrolyzable metal ions. *Water, Air, Soil Pollut.* **145**, 155-168, (2003).
3. N. Israelachvili, J. Intermolecular and Surface Forces. Ch. 11, Page 177, (Harcourt Brace & Company, 1991).
4. Huang, C., Zhao, X., Liu, S., Hao, Y., Tang, Q., Hu, A., Liu, Z. & Chen, X. Stabilizing zinc anodes by regulating the electrical double layer with saccharin anions. *Adv. Mater.* **33**, 2100445, (2021).
5. Sun, P., Ma, L., Zhou, W., Qiu, M., Wang, Z., Chao, D. & Mai, W. Simultaneous regulation on solvation shell and electrode interface for dendrite-free Zn ion batteries achieved by a low-cost glucose additive. *Angew. Chem., Int. Ed.* **60**, 18247-18255, (2021).
6. Li, Y., Wu, P., Zhong, W., Xie, C., Xie, Y., Zhang, Q., Sun, D., Tang, Y. & Wang, H.-Y. Progressive nucleation mechanism enables stable zinc stripping-plating behavior. *Energy Environ. Sci.* **14**, 5563-5571, (2021).
7. Zhu, M., Hu, J., Lu, Q., Dong, H., Karnaushenko, D. D., Becker, C., Karnaushenko, D., Li, Y., Tang, H., Qu, Z., Ge, J. & Schmidt, O. G. A patternable and in situ formed polymeric zinc blanket for a reversible zinc anode in a skin-mountable microbattery. *Adv. Mater.* **33**, e2007497, (2021).
8. Zeng, Y., Zhang, X., Qin, R., Liu, X., Fang, P., Zheng, D., Tong, Y. & Lu, X. Dendrite-free zinc deposition induced by multifunctional CNT frameworks for stable flexible Zn-ion batteries. *Adv. Mater.* **31**, 1903675, (2019).
9. Zhao, Z., Zhao, J., Hu, Z., Li, J., Li, J., Zhang, Y., Wang, C. & Cui, G. Long-life and deeply rechargeable aqueous Zn anodes enabled by a multifunctional brightener-inspired interphase. *Energy Environ. Sci.* **12**, 1938-1949, (2019).
10. Ma, L., Li, Q., Ying, Y., Ma, F., Chen, S., Li, Y., Huang, H. & Zhi, C. Toward practical high-areal-capacity aqueous zinc-metal batteries: quantifying hydrogen evolution and a solid-ion conductor for stable zinc anodes. *Adv. Mater.* **33**, e2007406, (2021).
11. Xie, X., Liang, S., Gao, J., Guo, S., Guo, J., Wang, C., Xu, G., Wu, X., Chen, G. & Zhou, J. Manipulating the ion-transference kinetics and interface stability for high-performance zinc metal anode. *Energy Environ. Sci.* **13**, 503-510, (2020).
12. Lee, D., Kim, H. I., Kim, W. Y., Cho, S. K., Baek, K., Jeong, K., Ahn, D. B., Park, S., Kang, S. J. & Lee, S. Y. Water-repellent ionic liquid skinny gels customized for aqueous Zn-ion battery anodes. *Adv. Funct. Mater.* **31**, 2103850, (2021).
13. Tian, H., Li, Z., Feng, G., Yang, Z., Fox, D., Wang, M., Zhou, H., Zhai, L., Kushima, A., Du, Y., Feng, Z., Shan, X. & Yang, Y. Stable, high-performance, dendrite-free, seawater-based aqueous batteries. *Nat. Commun.* **12**, 237, (2021).
14. Cui, Y., Zhao, Q., Wu, X., Chen, X., Yang, J., Wang, Y., Qin, R., Ding, S., Song, Y., Wu, J., Yang, K., Wang, Z., Mei, Z., Song, Z., Wu, H., Jiang, Z., Qian, G., Yang, L. & Pan, F. An interface-bridged organic-inorganic layer that suppresses dendrite formation and side reactions for ultra-long-life aqueous zinc metal anodes. *Angew. Chem., Int. Ed.* **59**, 16594-16601, (2020).
15. Zhang, Q., Luan, J., Huang, X., Wang, Q., Sun, D., Tang, Y., Ji, X. & Wang, H. Revealing the role of crystal orientation of protective layers for stable zinc anode. *Nat. Commun.* **11**, 3961, (2020).
16. Smith, A. M., Borkovec, M. & Trefalt, G. Forces between solid surfaces in aqueous electrolyte solutions. *Adv. Colloid Interface Sci.* **275**, 102078, (2020).
17. Derjaguin, B. & Landau, L. Theory of the Stability of Strongly Charged Lyophobic Sols and of the Adhesion of Strongly Charged Particles in Solutions of Electrolytes. *Prog. Surf. Sci.* **14**, 633-662, (1941).

18. Verwey, E. J. W. & Overbeek, J. T. G. Theory of the Stability of Lyophobic Colloids. Ch. 2, Pages 22-46, (Elsevier, 1948).
19. Elimelech., M., Xiadong, J., John, G. & Richard, W. Particle Deposition and Aggregation: Measurement, Modeling, and Simulation. Ch. 3, Pages 33-49, (Oxford: Butterworth-Heinemann Ltd., 1995).
20. Delgado, A. V., López-Viota, J., Ramos, M. M. & Arias, J. L. Colloid and interface science in pharmaceutical research and development. Ch. 21, Pages 443-460, (Amsterdam: Elsevier, 2014).
21. N. Israelachvili, J. Intermolecular and Surface Forces. Ch. 12, Page 238, (Harcourt Brace & Company, 1991).
22. N. Israelachvili, J. Intermolecular and Surface Forces. Ch. 12, Pages 241-248, (Harcourt Brace & Company, 1991).
23. N. Israelachvili, J. Intermolecular and Surface Forces. Ch. 12, Pages 213-259, (Harcourt Brace & Company, 1991).
24. Shohet, J. L. Encyclopedia of Physical Science and Technology. Page 412, (San Diego: Academic Press, 2003).
25. Omija, K., Hakim, A., Masuda, K., Yamaguchi, A. & Kobayashi, M. Effect of counter ion valence and pH on the aggregation and charging of oxidized carbon nanohorn (CNHox) in aqueous solution. *Colloids Surf., A* **619**, 126552, (2021).
26. PASHLEY, R. M. Forces between mica surfaces in La^{3+} and Cr^{3+} electrolyte solutions. *J. Colloid Interface Sci.* **102**, (1984).
27. Sabín, J., Prieto, G., Messina, P. V., Ruso, J. M., Hidalgo-Alvarez, R. & Sarmiento, F. On the effect of Ca^{2+} and La^{3+} on the colloidal stability of liposomes. *Langmuir* **21**, 10968-10975, (2005).
28. Kotynska, J. & Figaszewski, Z. A. Binding of trivalent metal ions (Al^{3+}, In^{3+}, La^{3+}) with phosphatidylcholine liposomal membranes investigated by microelectrophoresis. *Eur. Phys. J. E* **41**, 70, (2018).
